# Longitudinal Investigation into Genetics in the Conservation of Metabolic Phenotypes in Danish and Chinese Twins

**DOI:** 10.1371/journal.pone.0162805

**Published:** 2016-09-12

**Authors:** Shuxia Li, Kirsten Ohm Kyvik, Haiping Duan, Dongfeng Zhang, Zengchang Pang, Jacob Hjelmborg, Qihua Tan, Torben Kruse, Christine Dalgård

**Affiliations:** 1 Unit of Human Genetics, Department of Clinical Research, University of Southern Denmark, Odense, Denmark; 2 Department of Clinical Research, University of Southern Denmark, and Odense Patient data Explorative Network (OPEN), Odense University Hospital, Odense, Denmark; 3 Qingdao Center for Disease Control and Prevention, Qingdao, China; 4 Department of Public Health, Qingdao University Medical College, Qingdao, China; 5 Epidemiology, Biostatistics and Biodemography, Department of Public Health, University of Southern Denmark, Odense, Denmark; 6 Environmental Medicine, Department of Public Health, University of Southern Denmark, Odense, Denmark; University of Palermo, ITALY

## Abstract

Longitudinal twin studies on long term conservation of individual metabolic phenotypes can help to explore the genetic and environmental basis in maintaining metabolic homeostasis and metabolic health. We performed a longitudinal twin study on 12 metabolic phenotypes from Danish twins followed up for 12 years and Chinese twins traced for 7 years. The study covered a relatively large sample of 502 pairs of Danish adult twins with a mean age at intake of 38 years and a total of 181 Chinese adult twin pairs with a mean baseline age of 39.5 years. Bivariate twin models were fitted to the longitudinal measurements taken at two time points (at baseline and follow-up) to estimate the genetic and environmental contributions to phenotype variation and correlation at and between the two time points. High genetic components in the regulation of intra-individual phenotype correlation or stability over time were estimated in both Danish (h^2^>0.75 except fasting blood glucose) and Chinese (h^2^>0.72 except blood pressure) twins; moderate to high genetic contribution to phenotype variation at the two time points were also estimated except for the low genetic regulation on glucose in Danish and on blood pressure in Chinese twins. Meanwhile the bivariate twin models estimated shared environmental contributions to the variance and covariance in fasting blood glucose in Danish twins, and in systolic and diastolic blood pressure, low and high density lipoprotein cholesterol in Chinese twins. Overall, our longitudinal twin study on long-term stability of metabolic phenotypes in Danish and Chinese twins identified a common pattern of high genetic control over phenotype conservation, and at the same time revealed population-specific patterns of genetic and common environmental regulation on the variance as well as covariance of glucose and blood pressure.

## Introduction

Similar to most complex traits, metabolic phenotypes are regulated by both genetic and environmental factors with the interaction between them as central to the development of metabolic abnormality and diseases [1, 2]. Assessing the genetic and environmental basis in determining the longitudinal trajectory of metabolic traits can provide important information for exploring the roles of nature (an individual's innate qualities) and nurture (individual experience) in the development of metabolic phenotypes and for designing more efficient individualized strategy of healthcare, prevention and treatment.

The longitudinal pattern of metabolic traits can reflect both long-term stability and change in phenotype over time. Although the progression of metabolic phenotypes over life course can be more indicative of disorder-related modifications and disease onset [3], studying the long term conservation of personal metabolic phenotypes can help with exploring the genetic and environmental basis in maintaining metabolic homeostasis and metabolic health status. In the literature, the genetic and environmental contributions to metabolic phenotypes and metabolic diseases have been intensively studied using family [4–8] and twin [9–16] data. However, current studies are dominated by cross-sectional design that focuses on the level instead of longitudinal pattern of metabolic phenotypes perhaps due to difficulties in carrying out longitudinal experiments and in longitudinal data collection. As a result, only a limited number of studies have been published on the genetics in maintaining long-term stability of metabolic phenotypes. Among them, Franz et al. [17] reported that genetic factors explain 76% of phenotype correlation (genetic correlation of 0.6) between body mass index (BMI) at age 20 and age 48 in a cohort of male twins. Moderate genetic correlation on lipids levels (total cholesterol: 0.65; triglyceride: 0.73; low density lipoprotein cholesterol: 0.72; high density lipoprotein cholesterol: 0.45) measured over time was also estimated in adolescent twins during a seven years follow up [18]. Although focused on different traits in samples of different age groups, results from the two studies suggested important genetic contribution in stabilizing individual metabolic patterns over time. Recently, Yousri et al. [3] showed long term conservation patterns of human metabolites in adults which can be positively correlated with heritability estimates from a cross-sectional sample of twins. Although interesting, the currently published studies are either restricted to highly selected traits [17, 18] or make conclusions indirectly from different samples [3].

This paper reports results from two longitudinal studies on multiple metabolic phenotypes covering body mass, lipids, glucose and blood pressure conducted in Danish (followed up for 12 years) and Chinese (traced for 7 years) twins representing two ethnically distinct populations living in different social-cultural and geographic circumstances. The aim of the study is two-fold: firstly, to perform the first longitudinal twin study to assess the genetic and environmental contributions in maintaining metabolic homeostasis of different phenotypes in each of the two samples respectively; secondly, to identify and compare the different patterns of genetic regulation on the long-term stability of metabolic phenotypes between the two samples representing eastern (Chinese) and western (Danish) populations.

## Materials and Methods

### The Danish twins

Participants for the Danish study were recruited from the nationwide, population-based Danish Twin Registry during 1997–2000 to examine the genetic and environmental backgrounds in the development of insulin resistance, abdominal obesity and cardiovascular risk factors, i.e. the GEMINAKAR study as described previously [9–12]. Twins who consented to participation were followed up during 2010 to 2012. At the intake, the exclusion criteria included known diabetes or cardiovascular disease, conditions making a progressive maximal bicycle test impossible, pregnancy, and breast feeding. The cohort consisted of 756 complete twin pairs (783 females, 729 males, among them, 309 monozygotic (MZ) and 447 dizygotic (DZ) twin pairs) who underwent an extensive full day clinical examination of a variety of phenotypes. Co-twins had a clinical examination at the same day. The mean age of the participants at baseline was 38 years with a range of 18–67 [12]. A total of 1139 twins agreed to join the follow-up study of which 502 were complete pairs, hereof 226 MZ and 276 DZ pairs (among them 545 are females and 459 are males). The mean age at end of follow-up was 50 (range: 30–75) years.

Zygosity of twins was determined using microsatellite markers (AmpFISTR Profiler Plus Kit; PE Applied Biosystems, Perkin Elmer, Foster City, CA, USA). The Danish part of the study was approved by the local scientific committee of the Region of Southern Denmark (baseline, S-VF-19970271; follow-up, S-20090065) and by the Danish Data protection Board (baseline, 1999-1200-441; follow-up, 2009-41-2990). Written informed consent was obtained from all participants in the study.

### The Chinese twins

The sampling of the Chinese twins was based on the Qingdao Twin Registry at the Qingdao Center for Disease Control and Prevention (Qingdao CDC). At baseline, twins were recruited randomly through residence registry and the local disease control network of Qingdao CDC in 2006–2007. The exclusion criterion included pregnancy, breastfeeding, known diabetes and/or cardiovascular disease and use of weight-reducing medicaments within one month [14]. Only complete twin pairs who participated both investigations at baseline (time 1) and follow-up (time 2) were included. The same procedure for data collection was applied at both baseline and follow-up studies. A total of 181 complete twin pairs (101 MZ twin pairs and 80 DZ twin pairs, 245 female and 117 male twins) were identified with longitudinal measurements taken about 7 years apart with a mean age of 39.5 (range: 23–64) years at baseline and 46.5 (range: 30–71) years at end of follow-up. Zygosity of twins was identified using 16 short tandem repeat DNA markers at the central laboratory of Qingdao Blood Bank. The Chinese part of the study was approved by the local ethics committee at Qingdao CDC, Qingdao, China. Informed consent was obtained in writing from all participants.

### Metabolic phenotypes

The 12 metabolic phenotypes included total cholesterol (TC), triglycerides (TG), high density lipoprotein cholesterol (HDL), low density lipoprotein cholesterol (LDL), fasting blood glucose (GLU), body weight (WT), body mass index (BMI), waist (WAIST), hip (HIP) circumference, waist-hip- ratio (WHR), systolic (SBP), and diastolic (DBP) blood pressure. All metabolic phenotypes were measured according to standard procedures as described elsewhere for both samples [12, 14]. In briefly, body weight was measured using a standing beam scale and to the nearest 0.1 kilogram (kg) and height measured using a vertical scale with a horizontal moving headboard and to the nearest centimeter (cm). Waist and hip circumferences (in cm) were taken in standing position with waist circumference measured midway between the lowest rib and the iliac crest, and hip circumference measured over the widest part of the gluteal region. BMI was calculated as weight (kg) divided by the square of height in meter (m^2^). SBP and DBP measurements (mmHg) were taken after at least 5 minutes of rest following a standard procedure using a conventional mercurial sphygmomanometer. Three measurements were taken from each subject, with at least 1 minute between each measurement and the mean hereof was calculated and used in subsequent analyses. GLU concentration was analyzed by the glucose dehydrogenase oxidation method both for Danish and Chinese blood samples [9, 14]. Blood lipids levels were measured following standard laboratory protocols for both Danish (LDL was calculated from TC, HDL and TG using Friedewald formula) and Chinese twins [11, 14].

### Analysis of twin data

**Fig 1:**
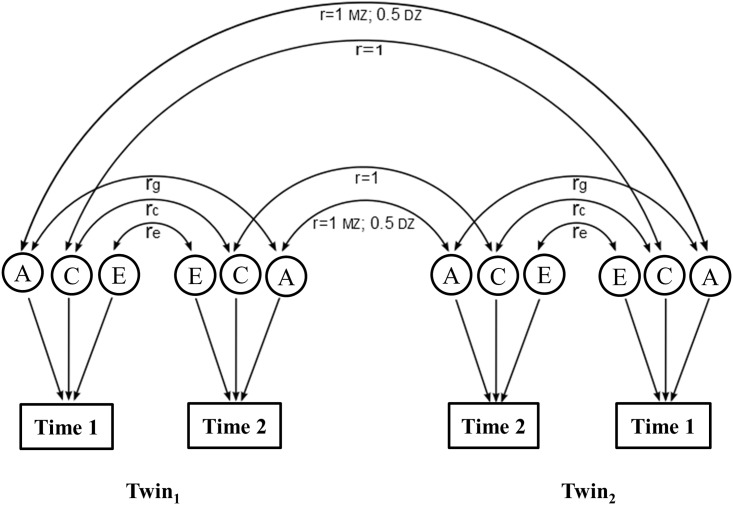
Path diagram of the bivariate twin model applied to longitudinal data assuming additive genetic (A), common environmental (C), and unique environmental (E) components in the variances and covariance of phenotype measurements at time 1 (baseline) and time 2 (follow-up). The genetic correlation is set 1.0 in MZ twin pairs and 0.5 in DZ twin pairs. The shared environmental correlations is set to 1.0 in all twin pairs; *r*_*g*_, *r*_*c*_, *r*_*e*_ are the genetic, shared environmental and unique environmental correlations on phenotype levels at the two time points, respectively. Hereof *r*_*g*_ can be calculated as $r_{g}=\frac{{\text{cov}}_{g}(\text{Time }1,\text{Time }2)}{\sqrt{{\text{var}}_{g}(\text{Time }1){\text{var}}_{g}(\text{Time }2)}}$, where var_g_ (Time 1), var_g_ (Time 2) are the additive genetic variance of Time 1 and Time 2 respectively, and cov_g_ (Time 1,Time 2) is their genetic covariance. Likewise, correlations for common environmental (*r*_*c*_), and unique environmental (*r*_*e*_) factors can be calculated.

The classical twin method was applied to analyze the longitudinal data by fitting bivariate twin models (Fig 1) to the measurements at time 1 (baseline) and time 2 (follow-up) to estimate the genetic and environmental components in the phenotype variance at the each time point and in the phenotype covariance between the two times. The bivariate twin model was primarily used to assess genetic pleiotropy between two phenotypes [19]. The same idea has been subsequently applied to longitudinal twin data to estimate the genetic correlation on levels of one phenotype measured over time on same individuals [17, 18]. Using the typical structural equation modeling (SEM) approach [20], the bivariate twin model decomposed the total phenotype variance and covariance at and between the two waves into additive genetic (A), common or shared environmental (C), and unique environmental (E) components in fitting univariate and bivariate twin models. Based on the full ACE model, nested models were also fitted by dropping the C (AE model), the A (CE model), or both (E model) components for best model selection. Performances between the full models and its nested models were compared using the likelihood ratio test (LRT) for best fitting model selection. The parsimonious model was preferred than the full model when no statistical significance is observed between them. Goodness of fit was assessed by calculating the Akaike Information Criterion (AIC) [21]. From models fitted with a genetic component A, heritability (*h*^*2*^) can be calculated as the proportion of genetic variance among the total variance, i.e. $h^{2}=\frac{A}{A+C+E}$ for the ACE model and $h^{2}=\frac{A}{A+E}$ for the AE model.

For each parameter estimated, a 95% confidence interval (CI) was computed using the likelihood-based method [22]. As described by Neale and Miller [22], the likelihood-based confidence intervals have superior statistical properties to the more common type based on derivatives (standard errors) mainly because the parameter estimates may not follow normal distribution. In all the analyses, age and sex were taken as co-variants in the models for adjustment.

Before fitting the twin models, each phenotype value was log transformed to minimize possible skewed phenotype distribution. The free Mx package (http://www.vcu.edu/mx) was used for fitting bivariate models.

## Results

Table 1 shows the means of the 12 phenotypes at baseline and at follow-up and intra-individual phenotype correlation (r_1,2_) with its 95% confidence interval between the two waves for both Danish and Chinese twins. The overall mean of individual phenotypes varied with waves, meanwhile we also found significant intra-individual phenotype correlation between the two time points for each of the 12 phenotypes and in each of the two samples providing the basis for exploring the genetic and environmental components in the maintaining phenotypic stability.

**Table 1:** Phenotype means and intra-individual phenotype correlation between wave 1 and wave 2 in Danish and Chinese twins.

	Danish Twins				Chinese			
Traits	Mean, 1	Mean, 2	r_1,2_	95% CIs	Mean, 1	Mean, 2	r_1,2_	95% CIs
**TC, mmol/L**	5.36	5.48	0.52	0.45–0.58	5.26	4.91	0.64	0.54–0.72
**TG, mmol/L**	1.27	1.23	0.51	0.45–0.58	1.18	1.25	0.34	0.20–0.46
**HDL, mmol/L**	1.52	1.55	0.56	0.50–0.62	1.57	1.57	0.42	0.29–0.53
**LDL, mmol/L**	3.29	3.37	0.53	0.46–0.59	3.10	2.68	0.53	0.41–0.63
**GLU, mmol/L**	4.76	5.58	0.46	0.39–0.53	4.71	5.42	0.72	0.64–0.78
**WT, kg**	73.18	76.59	0.88	0.86–0.90	62.70	64.03	0.93	0.91–0.95
**BMI, kg/m**^**2**^	24.43	25.73	0.82	0.79–0.85	23.89	24.45	0.91	0.88–0.93
**WAIST, cm**	83.77	88.04	0.75	0.70–0.78	77.32	81.94	0.80	0.72–0.85
**HIP, cm**	96.40	102.17	0.57	0.51–0.63	96.82	96.92	0.78	0.70–0.84
**WHR**	0.87	0.86	0.64	0.59–0.69	0.80	0.84	0.66	0.54–0.75
**SBP, mmHg**	116.36	123.43	0.55	0.49–0.61	118.11	125.38	0.60	0.47–0.70
**DBP, mmHg**	68.16	79.42	0.46	0.38–0.52	80.36	81.55	0.58	0.45–0.69

We next fitted full bivariate twin models to the phenotype measurements at the two times. As described in the method section, nested model were also fitted by dropping the additive genetic and shared environmental components in the phenotypic variances at time 1 (baseline) and time 2 (follow-up) and in the phenotypic covariance between them. After comparison in performances of all nested models with corresponding full model of each phenotype, the best fitting model was selected (Tables 2 and 3) which all outperformed the full models with no significant difference in goodness of fit between the full and best-fitting model (last column in Tables 2 and 3). Table 4 presents the best fitting models and their parameter estimates with 95% CIs for the Danish twins. Except for GLU, all other phenotypes had very high proportions of additive genetic covariance ranging from 0.75 (95% CI: 0.67–0.75) for WAIST to 0.95 (95% CI: 0.86–1.00) for LDL. The best model for GLU had no genetic component in its covariance. Instead, the covariance for GLU at the two time points was mostly determined by the shared environmental factors (0.72, 95% CI: 0.58–0.86). This was different from the other metabolic phenotypes which had no estimated common environmental contribution to their intra-individual phenotypic correlation over time. In addition, the best fitting models also estimated moderate to high additive genetic components in phenotype variation at each of the two time points except for GLU (low estimates of A: 0.14 at time 1 and 0.25 at time 2). All phenotypes had low to moderate E components in phenotype variation at the two times and low E components in intra-individual phenotype covariance between the two times.

**Table 2:** Models comparison between full and nested models in Danish twins (best fitting model marked with an asterisk).

Phenotypes	models	AIC	Δdf	Χ^2^	P values
**TC**	ACE	-5300.41			
	No r_g_ ACE	-5269.47	1	32.94	0.00
	No r_c_ ACE	-5302.41	1	0.00	1.00
	No r_e_ ACE	-5297.41	1	5.00	0.03
	AE*	**-5306.41**	**3**	**0.00**	**1.00**
	CE	-5217.55	3	88.86	0.00
**TG**	ACE	-2150.60			
	No r_g_ ACE	-2127.75	1	24.85	0.00
	**No r**_**c**_ ACE*	**-2151.52**	**1**	**1.08**	**0.30**
	No r_e_ ACE	-2135.69	1	16.91	0.00
	AE	-2149.73	3	6.87	0.08
	CE	-2131.01	3	25.60	0.00
**HDL**	ACE	-4370.18			
	No r_g_ ACE	-4333.94	1	38.24	0.00
	**No r**_**c**_ ACE*	**-4372.11**	**1**	**0.07**	**0.79**
	No r_e_ ACE	-4364.85	1	7.33	0.01
	AE	-4364.18	3	12.00	0.01
	CE	-4316.16	3	60.05	0.00
**LDL**	ACE	-3475.01			
	No r_g_ ACE	-3439.43	1	37.58	0.00
	No r_c_ ACE	-3476.96	1	0.14	0.84
	No r_e_ ACE	-3476.16	1	0.88	0.35
	AE*	**-3480.87**	**3**	**0.14**	**0.99**
	CE	-3422.84	3	58.16	0.00
**GLU**	ACE	-7683.57			
	**No r**_**g**_ ACE*	**-7684.65**	**1**	**0.92**	**0.34**
	No r_c_ ACE	-7678.22	1	7.36	0.01
	No r_e_ ACE	-7680.45	1	5.13	0.02
	AE	-7681.88	3	7.69	0.05
	CE	-7676.29	3	13.29	0.00
**WT**	ACE	-7447.93			
	No r_g_ ACE	-7399.70	1	50.23	0.00
	No r_c_ ACE	-7449.65	1	0.28	0.59
	No r_e_ ACE	-7286.90	1	163.03	0.00
	AE*	**-7453.65**	**3**	**0.28**	**0.96**
	CE	-7391.30	3	62.63	0.00
**BMI**	ACE	-7617.55			
	No r_g_ ACE	-7577.26	1	42.29	0.00
	No r_c_ ACE	-7618.24	1	1.32	0.25
	No r_e_ ACE	-7482.38	1	137.17	0.00
	AE*	**-7622.24**	**3**	**1.32**	**0.73**
	CE	-7569.70	3	53.85	0.00
**WAIST**	ACE	-8005.42			
	No r_g_ ACE	-7983.46	1	23.97	0.00
	No r_c_ ACE	-8007.27	1	0.16	0.69
	No r_e_ ACE	-7935.93	1	71.50	0.00
	AE*	**-8011.27**	**3**	**0.16**	**0.98**
	CE	-7968.88	3	42.54	0.00
**HIP**	ACE	-9066.68			
	No r_g_ ACE	-9034.85	1	33.83	0.00
	**No r**_**c**_ ACE*	**-9068.46**	**1**	**0.23**	**0.64**
	No r_e_ ACE	-9032.73	1	35.95	0.00
	AE	-9060.07	3	12.61	0.01
	CE	-9028.15	3	44.54	0.00
**WHR**	ACE	-8624.15			
	No r_g_ ACE	-8612.79	1	13.37	0.00
	**No r**_**c**_ ACE*	**-8625.99**	**1**	**0.17**	**0.68**
	No r_e_ ACE	-8618.57	1	7.58	0.00
	AE	-8618.63	3	11.53	0.01
	CE	-8592.85	3	37.31	0.00
**SBP**	ACE	-7913.84			
	No r_g_ ACE	-7892.84	1	23.00	0.00
	No r_c_ ACE	-7915.84	1	0.00	1.00
	No r_e_ ACE	-7914.84	1	1.01	0.32
	AE*	**-7919.72**	**3**	**0.12**	**0.99**
	CE	-7874.11	3	45.73	0.00
**DBP**	ACE	-7231.87			
	No r_g_ ACE	-7221.73	1	12.14	0.00
	No r_c_ ACE	-7233.18	1	0.69	0.41
	No r_e_ ACE	-7230.65	1	3.22	0.07
	AE*	**-7236.97**	**3**	**0.90**	**0.82**
	CE	-7199.10	3	38.77	0.00

**Table 3:** Models comparison between full and nested models in Chinese twins (best fitting model marked with an asterisk).

Phenotypes	models	AIC	Δdf	Χ^2^	P values
**TC**	ACE	-1989.69			
	No r_g_ ACE	-	1	-	-
	No r_c_ ACE	-1991.16	1	0.52	0.47
	No r_e_ ACE	-1989.77	1	1.92	0.17
	AE*	**-1993.12**	**3**	**2.57**	**0.46**
	CE	-	3	-	-
**TG**	ACE	-378.01			
	No r_g_ ACE	-	1	-	-
	No r_c_ ACE	-379.09	1	0.92	0.34
	No r_e_ ACE	-376.70	1	3.31	0.07
	AE*	**-380.23**	**3**	**3.78**	**0.29**
	CE	-	3	-	-
**HDL**	ACE	-1708.53			
	No r_g_ ACE	-1700.64	1	9.90	0.00
	No r_c_ ACE	-1709.27	1	1.26	0.26
	**No r**_**e**_ ACE*	**-1710.39**	**1**	**0.14**	**0.71**
	AE	-1697.77	3	16.77	0.00
	CE	-1692.21	3	22.33	0.00
**LDL**	ACE	-1725.92			
	No r_g_ ACE	-1713.06	1	14.90	0.00
	No r_c_ ACE	-1727.54	1	0.38	0.54
	**No r**_**e**_ ACE*	**-1727.82**	**1**	**0.11**	**0.75**
	AE	-1718.72	3	13.20	0.00
	CE	-1704.66	3	27.26	0.00
**GLU**	ACE	-2140.92			
	No r_g_ ACE	-2112.96	1	29.96	0.00
	No r_c_ ACE	-2142.43	1	0.49	0.49
	No r_e_ ACE	-2142.85	1	0.07	0.79
	AE*	**-2142.88**	**3**	**4.04**	**0.26**
	CE	-2116.20	3	30.72	0.00
**WT**	ACE	-2833.25			
	No r_g_ ACE	-2769.56	1	65.69	0.00
	No r_c_ ACE	-2835.15	1	0.10	0.75
	No r_e_ ACE	-2745.86	1	89.40	0.00
	AE*	**-2836.93**	**3**	**2.32**	**0.51**
	CE	-2770.20	3	69.05	0.00
**BMI**	ACE	-2965.65			
	No r_g_ ACE	-2925.11	1	42.54	0.00
	No r_c_ ACE	-2967.65	1	0.00	0.98
	No r_e_ ACE	-2900.39	1	67.26	0.00
	AE*	**-2968.26**	**3**	**3.39**	**0.34**
	CE	-2923.53	3	48.12	0.00
**WAIST**	ACE	-2290.97			
	No r_g_ ACE	-2270.95	1	22.01	0.00
	**No r**_**c**_ ACE*	**-2292.73**	**1**	**0.23**	**0.63**
	No r_e_ ACE	-2282.81	1	83.31	0.00
	AE	-2291.02	3	5.95	0.11
	CE	-2271.33	3	25.64	0.00
**HIP**	ACE	-3001.92			
	No r_g_ ACE	-2983.20	1	20.7	0.00
	**No r**_**c**_ ACE*	**-3003.47**	**1**	**0.45**	**0.50**
	No r_e_ ACE	-2978.46	1	25.46	0.00
	AE	-3002.71	3	5.21	0.16
	CE	-2984.43	3	23.50	0.00
**WHR**	ACE	-2724.99			
	No r_g_ ACE	-2712.24	1	14.74	0.00
	**No r**_**c**_ ACE*	**-2726.89**	**1**	**0.10**	**0.75**
	No r_e_ ACE	-2726.01	1	0.98	0.32
	AE	-2721.91	3	9.08	0.03
	CE	-2712.12	3	18.87	0.00
**SBP**	ACE	-2133.27			
	No r_g_ ACE	-2130.07	1	5.20	0.02
	No r_c_ ACE	-2127.79	1	7.48	0.01
	**No r**_**e**_ ACE*	**-2135.27**	**1**	**0.00**	**0.96**
	AE	-2129.69	3	9.58	0.02
	CE	-2130.86	3	8.41	0.04
**DBP**	ACE	-2150.98			
	No r_g_ ACE	-2149.62	1	3.36	0.07
	No r_c_ ACE	-2145.67	1	7.31	0.01
	**No r**_**e**_ ACE*	**-2152.60**	**1**	**0.38**	**0.54**
	AE	-2143.66	3	13.32	0.00
	CE	-2151.05	3	5.93	0.12

**Table 4:** Parameter estimates and 95% CIs for the best fitting bivariate models for Danish twins.

Traits	Time 1			Time 2			Covariance			Correlation		
	a^2^	c^2^	e^2^	a^2^	c^2^	e^2^	a^2^	c^2^	e^2^	r^g^	r^c^	r^e^
**TC**	0.78 (0.73–0.82)		0.22 (0.18–0.27)	0.60 (0.52–0.67)		0.40 (0.33–0.48)	0.91 (0.81–0.99)		0.09 (0.01–0.19)	0.58 (0.49–0.66)		0.14 (0.02–0.26)
**TG**	0.45 (0.28–0.60)	0.08 (0.00–0.22)	0.47 (0.39–0.56)	0.34 (0.21–0.51)	0.12 (0.00–0.22)	0.54 (0.46–0.62)	0.78 (0.65–0.88)		0.22 (0.12–0.35)	1.00 (0.80–1.00)		0.22 (0.12–0.32)
**HDL**	0.32 (0.24–0.51)	0.30 (0.14–0.36)	0.38 (0.32–0.45)	0.65 (0.57–0.72)	0.00 (0.00–0.05)	0.35 (0.28–0.43)	0.89 (0.79–0.97)		0.11 (0.03–0.21)	1.00 (0.78–1.00)		0.15 (0.04–0.27)
**LDL**	0.65 (0.58–0.71)		0.35 (0.29–0.42)	0.66 (0.58–0.72)		0.34 (0.28–0.42)	0.95 (0.86–1.00)		0.05 (0.00–0.14)	0.71 (0.62–0.80)		0.07 (0.00–0.19)
**GLU**	0.14 (0.00–0.31)	0.32 (0.18–0.47)	0.54 (0.46–0.63)	0.25 (0.08–0.38)	0.20 (0.11–0.32)	0.55 (0.46–0.65)		0.72 (0.58–0.86)	0.28 (0.14–0.42)		1.00 (0.80–1.00)	0.18 (0.08–0.27)
**WT**	0.76 (0.71–0.80)		0.24 (0.20–0.29)	0.72 (0.66–0.77)		0.28 (0.23–0.34)	0.81 (0.75–0.85)		0.19 (0.15–0.25)	0.93 (0.90–0.95)		0.63 (0.55–0.70)
**BMI**	0.73 (0.68–0.78)		0.27 (0.22–0.32)	0.71 (0.65–0.76)		0.29 (0.24–0.35)	0.80 (0.74–0.85)		0.20 (0.15–0.25)	0.92 (0.89–0.95)		0.59 (0.51–0.66)
**WAIST**	0.66 (0.66–0.72)		0.34 (0.28–0.36)	0.60 (0.59–0.66)		0.40 (0.34–0.41)	0.75 (0.67–0.75)		0.25 (0.25–0.33)	0.81 (0.75–0.82)		0.45 (0.45–0.54)
**HIP**	0.59 (0.46–0.64)	0.17 (0.09–0.28)	0.24 (0.22–0.28)	0.51 (0.46–0.57)	0.13 (0.11–0.14)	0.36 (0.31–0.36)	0.84 (0.81–0.86)		0.16 (0.14–0.19)	0.92 (0.83–0.93)		0.33 (0.26–0.42)
**WHR**	0.38 (0.20–0.57)	0.30 (0.24–0.45)	0.32 (0.27–0.39)	0.51 (0.37–0.57)	0.00 (0.00–0.12)	0.49 (0.43–0.57)	0.79 (0.63–0.93)		0.21 (0.07–0.32)	0.57 (0.57–0.75)		0.17 (0.06–0.25)
**SBP**	0.68 (0.61–0.73)		0.32 (0.27–0.39)	0.51 (0.42–0.59)		0.49 (0.41–0.58)	0.94 (0.82–1.00)		0.06 (0.00–0.18)	0.67 (0.56–0.77)		0.07 (0.00–0.19)
**DBP**	0.65 (0.60–0.71)		0.35 (0.29–0.40)	0.55 (0.46–0.55)		0.45 (0.45–0.54)	0.89 (0.75–1.00)		0.11 (0.00–0.25)	0.55 (0.44–0.66)		0.11 (0.00–0.23)

The Chinese data were analyzed in the same way as Danish twins. Table 5 shows the parameter estimates for the best fitting models for each phenotype. Except for blood pressure, all other phenotypes had very high proportions of genetic component in phenotype covariance between the two time points ranging from 0.72 for HDL to 1.00 for GLU. The bivariate models also estimated moderate to high proportions of genetic component in the phenotype variation at each of the 2 time points, again except for blood pressure. In addition to the genetic effects, the shared environment also had low to moderate contribution to the variance and covariance of the two times for lipoprotein (HDL, LDL) and blood pressure (SBP, DBP). Similar to the Danish twins, all phenotypes had low to moderate unique environmental contribution to their total variance at the two times and low E components in their covariance with no unique environmental covariance estimated for lipoproteins (LDL, HDL) and blood pressure (SBP, DBP).

**Table 5:** Parameter estimates and 95% CIs for the best fitting bivariate models for Chinese twins.

Traits	Time 1			Time 2			Covariance			Correlation		
	a^2^	c^2^	e^2^	a^2^	c^2^	e^2^	a^2^	c^2^	e^2^	r^g^	r^c^	r^e^
**TC**	0.80 (0.73–0.85)		0.20 (0.15–0.27)	0.77 (0.68–0.83)		0.23 (0.17–0.32)	0.95 (0.87–1.00)		0.05 (0.00–0.13)	0.75 (0.66–0.83)		0.15 (0.00–0.32)
**TG**	0.61 (0.50–0.70)		0.39 (0.30–0.49)	0.78 (0.70–0.84)		0.22 (0.16–0.30)	0.93 (0.80–1.00)		0.07 (0.00–0.19)	0.67 (0.69–0.75)		0.13 (0.00–0.30)
**HDL**	0.38 (0.19–0.67)	0.44 (0.16–0.63)	0.18 (0.13–0.24)	0.32 (0.09–0.64)	0.45 (0.14–0.66)	0.23 (0.17–0.32)	0.72 (0.33–1.00)	0.28 (0.00–0.67)		0.85 (0.42–1.00)	0.25 (0.00–0.62)	
**LDL**	0.47 (0.22–0.74)	0.28 (0.02–0.52)	0.25 (0.18–0.33)	0.47 (0.25–0.76)	0.21 (0.00–0.44)	0.32 (0.23–0.41)	0.87 (0.50–1.00)	0.13 (0.00–0.50)		1.00 (0.69–1.00)	0.28 (0.00–1.00)	
**GLU**	0.68 (0.58–0.75)		0.32 (0.25–0.42)	0.72 (0.64–0.79)		0.28 (0.21–0.36)	1.00 (0.95–1.00)		0.00 (0.00–0.05)	0.96 (0.89–1.00)		0.00 (0.00–0.11)
**WT**	0.86 (0.80–0.90)		0.14 (0.14–0.20)	0.83 (0.76–0.87)		0.17 (0.13–0.24)	0.88 (0.82–0.89)		0.12 0.09–0.18	0.96 (0.93–0.97)		0.71 (0.60–0.79)
**BMI**	0.83 (0.77–0.89)		0.17 (0.12–0.23)	0.77 (0.69–0.83)		0.23 (0.17–0.31)	0.86 (0.79–0.91)		0.14 (0.09–0.21)	0.95 (0.92–0.97)		0.63 (0.50–0.73)
**WAIST**	0.68 (0.50–0.81)	0.05 (0.00–0.22)	0.27 (0.18–0.38)	0.55 (0.39–0.76)	0.19 (0.00–0.32)	0.26 (0.19–0.36)	0.87 (0.76–0.95)		0.13 (0.05–0.24)	1.00 (0.84–1.00)		0.35 (0.14–0.52)
**HIP**	0.62 (0.48–0.80)	0.09 (0.00–0.20)	0.29 (0.20–0.41)	0.62 (0.43–0.76)	0.08 (0.00–0.23)	0.30 (0.23–0.41)	0.80 (0.67–0.89)		0.20 (0.12–0.33)	1.00 (0.87–1.00)		0.54 (0.36–0.67)
**WHR**	0.67 (0.42–0.80)	0.04 (0.00–0.27)	0.29 (0.20–0.42)	0.34 (0.20–0.59)	0.39 (0.16–0.52)	0.27 (0.20–0.36)	0.94 (0.79–1.00)		0.06 (0.00–0.21)	1.00 (0.71–1.00)		0.12 (0.00–0.33)
**SBP**	0.18 (0.03–0.48)	0.54 (0.25–0.70)	0.28 (0.19–0.38)	0.39 (0.12–0.63)	0.26 (0.04–0.52)	0.35 (0.26–0.46)	0.42 (0.13–0.82)	0.58 (0.18–0.87)		1.00 (0.66–1.00)	1.00 (0.69–1.00)	
**DBP**	0.12 (0.01–0.39)	0.60 (0.34–0.74)	0.28 (0.20–0.38)	0.42 (0.11–0.64)	0.21 (0.02–0.49)	0.37 (0.28–0.49)	0.41 (0.09–0.83)	0.59 (0.17–0.91)		1.00 (0.52–1.00)	0.92 (0.52–1.00)	

## Discussion

Based on longitudinal data from Danish and Chinese twins, we were able to assess the genetic and environmental basis in maintaining intra-individual stability of 12 metabolic phenotypes. Although the samples were taken from two populations of distinct ethnic and environmental background, we found consistent patterns of high genetic and low environmental controls over stability of nearly all metabolic phenotypes. Our results emphasize the high importance of genetic factors in the conservation of metabolic phenotypes.

In a recent study, Yousri et al. [3] analyzed long-term conservation of human metabolites and reported that metabolites displaying high intra-individual longitudinal correlation tended to show high genetic control on the level of metabolites. In Table 1, the intra-individual phenotype correlation was estimated for each of the 12 metabolic phenotypes with body mass traits exhibiting the highest intra-individual longitudinal correlation both in Danish and in Chinese twins. Indeed, as shown in Tables 4 and 5, the body mass traits tend to have high heritability estimates at each of the two time points, a phenomenon that supports the observation by Yousri et al. [3]. However, as it is shown in Tables 4 and 5, the estimated genetic components in covariance between the two times for body mass traits were not higher than that for the other metabolic phenotypes. Results from bivariate twin modeling suggest that high longitudinal phenotypic correlation does not necessarily mean high genetic control on phenotype stability over time.

In a recent analysis of the same data used in this paper, classical twin models were fitted to intra-individual longitudinal change (i.e. Δphenotype = phenotypewave2—phenotype_wave1_) instead of stability of metabolic phenotypes [23]. Contrary to the current study, the change of phenotype i.e. Δphenotype was predominantly controlled by the individual unique environment while the genetic contribution was very limited. The high genetic correlation on metabolic phenotypes between two time points estimated from current study is in contrast to the predominant control by the unique environment over longitudinal change of phenotype. The phenomenon emphasizes the high genetic involvement in maintaining metabolic phenotype stability. In fact, the ability in maintaining metabolic stability is an indispensable attribute of living cells that must have arisen with life’s origin and is necessary for conserving a stable intra-cellular environment (homeostasis) which is essential for maintaining an efficient functional state [24]. The high genetic correlation (Tables 4 and 5) between the phenotype measurements at the two waves suggested strong overlap in genes that regulate the phenotype level. In Danish twins (Table 4), complete longitudinal genetic correlation (r_g_ = 1) was estimated for TG, HDL and high genetic correlation (r_g_>0.9) for 3 out of the 5 body mass traits. In the Chinese twins (Table 5), high (r_g_>0.9) and complete genetic correlations were estimated for LDL, GLU, all body mass traits and blood pressure. It is interesting to see that, while there was a persistent genetic control over glucose metabolism in the Chinese twins, the best fitting model for GLU estimated no genetic correlation in the Danish twins (Table 4) who, instead, had complete common environmental correlation (r_c_ = 1). Moreover, the genetic contributions to GLU level at the two time points were all lower in Danish than in Chinese twins (at time 1: 0.14 versus 0.68 and time 2: 0.25 versus 0.72) (Tables 4 and 5), a phenomenon that was also reported in a cross-sectional twin study by Li et al. [25]. Taking together, we could conclude that the Chinese twins perhaps had high and lasting genetic regulation on glucose metabolism while in Danish twins might have differential genetic involvement in regulating the level of GLU at the two time points with the shared environment playing an important role in maintaining the blood glucose level over time. We may then postulate that the variation and stability in glucose concentration could be more controlled by genetic factors in Chinese twins but more by environmental factors in Danish twins. More work is needed to test and to validate our hypothesis.

In the Chinese twins, complete genetic correlation (r_g_ = 1) was estimated for SBP and DBP respectively, but corresponding proportion of genetic covariance was only moderate (0.42, 95% CI: 0.13–0.82 for SBP; 0.41, 95% CI: 0.09–0.83 for DBP). In Danish twins, however, the genetic correlation (r_g_) was only 0.67 for SBP and 0.55 for DBP but the corresponding proportion of genetic covariance was high for both SBP (0.94, 95% CI: 0.82–1.00) and DBP (0.89, 95% CI: 0.75–1.00). Based on the higher genetic correlation but lower proportion of genetic covariance in Chinese twins as compared with the lower genetic correlation but higher proportion of genetic covariance in the Danish twins, we assume that the genetic components are lasting/persistent over time but with limited/moderate contribution to blood pressure conservation in Chinese twins. In Danish twins, the high genetic covariance but low genetic correlation between the two times as compared to the Chinese twins lead to the assumption of a low degree of overlap in genes affecting blood pressure at each time point in the Danish twins although genetic factor plays an important role in maintaining long-term blood pressure stability. Moreover, apart from the complete genetic correlation, the Chinese twins also had very high shared environmental correlation on SBP (r_c_ = 1, 95% CI: 0.69–1.00) and on DBP (r_c_ = 0.92; 95% CI: 0.52–1.00) which was in striking difference with the Danish twins who had no estimates on common environment. In brief, both genetic and family environmental factors persistently contributed in maintaining the stability of SBP and of DBP in Chinese twins; the Danish twins displayed high genetic regulation over the levels of blood pressure, a pattern consistent with Li et al. [26].

In summary, our longitudinal twin study on long-term stability of metabolic phenotypes in Danish and Chinese twins identified a common pattern of high genetic control over metabolic phenotype conservation, and meanwhile revealed population-specific patterns of genetic and common environmental regulation over the variance as well as covariance of fasting blood glucose and blood pressure.
